# Notch signaling as a master regulator of adult neurogenesis

**DOI:** 10.3389/fnins.2023.1179011

**Published:** 2023-06-29

**Authors:** Aikaterini Lampada, Verdon Taylor

**Affiliations:** Department of Biomedicine, University of Basel, Basel, Switzerland

**Keywords:** Notch, neural stem cells, neurogenesis, subventricular zone, dentate gyrus

## Abstract

Neurogenesis ceases in most regions of the mammalian brain before or shortly after birth, however, in a few restricted brain regions, the production of new neurons proceeds into adulthood. Neural stem cells (NSCs) in these neurogenic zones are integrated into niches that control their activity and fate. Most stem cells in the adult brain are mitotically inactive and these cells can remain quiescent for months or even years. One of the key questions is what are the molecular mechanisms that regulate NSC maintenance and differentiation. Notch signaling has been shown to be a critical regulator of stem cell activity and maintenance in many tissues including in the nervous system. In this mini-review we discuss the roles of Notch signaling and the functions of the different Notch receptors and ligands in regulating neurogenesis in the adult murine brain. We review the functions of Notch signaling components in controlling NSC quiescence and entry into cell cycle and neurogenesis.

## Introduction

The mammalian brain is formed during embryogenesis and early postnatal life, with most neurons being born before birth. However, in the 1960s, the field of neurogenesis was revolutionized by the discovery that neurons are continually generated in distinct regions of the adult mammalian brain including in the cerebral cortex and the hippocampus (Altman, 1962; Altman and Das, 1965; Obernier and Alvarez-Buylla, 2019). Hitherto, it is widely accepted that neurogenesis occurs throughout life in two specialized niches of the adult rodent brain, the ventricular-subventricular zone (V-SVZ) and the dentate gyrus (DG) of the hippocampus (Goncalves et al., 2016; Obernier and Alvarez-Buylla, 2019). Adult neurogenesis has been documented in the brains of many vertebrate species including fish, rodents, birds and primates and, although there is some controversy in the field, evidence suggests the production of neurons also in brains of adult humans (Boldrini et al., 2018; Kempermann et al., 2018; Sorrells et al., 2018).

Adult neural stem cells (NSCs) are the source of new neurons during adult neurogenesis. NSCs are specialized radial glia (RG) that reside in dedicated niches in the walls of the lateral ventricles (LV) forming the V-SVZ, and in the subgranular zone (SGZ) of the DG. V-SVZ NSCs give-rise to different interneuron populations that migrate to the olfactory bulb (OB) and the SGZ NSCs generate granule cells in the DG (Doetsch et al., 1999; Seri et al., 2001). NSCs in both adult neurogenic niches express glial markers and are maintained in a quiescent state. Once activated, quiescent NSCs (qNSCs) enter the cell cycle and become active NSCs (aNSCs). Several NSC intrinsic and extrinsic factors have been shown to regulate adult neurogenesis through altering the equilibrium between signals that maintain quiescence and those that induce NSC activation (Berg et al., 2013; Tong et al., 2014; Matsubara et al., 2021). This equilibrium between quiescence and activation defines the rate of neurogenesis as well as the long-term maintenance of NSCs and neurogenesis in the brain niches (Bonaguidi et al., 2011; Encinas et al., 2011; Calzolari et al., 2015; Urban et al., 2016, 2019; Basak et al., 2018; Obernier et al., 2018; Pilz et al., 2018). Cell death of newborn neurons is also an important regulatory mechanism controlling the generation of mature neurons in the adult mouse brain (Sierra et al., 2010; Ryu et al., 2016; Pfisterer and Khodosevich, 2017).

In this review, we focus on the role of Notch signaling in the control of NSC activity and neurogenesis and address some of the unanswered questions about how this pathway plays different roles in the generation of neurons in the adult brain.

## NSCs of the adult neurogenic niches

NSCs in the adult V-SVZ and the SGZ niches share many similarities, however, they also have important differences in the organization, location and architecture of their niches and in their fate potentials that will be discussed below [for reviews see (Goncalves et al., 2016; Obernier and Alvarez-Buylla, 2019; Urban et al., 2019)].

## NSCs of the V-SVZ niche

NSCs in the adult V-SVZ (B1 cells) are generated by RGs during embryonic development. The majority of the V-SVZ NSC precursors are generated as early as embryonic day 13.5 and remain quiescent through the late stages of embryogenesis until postnatal and adult stages of life (Merkle et al., 2004; Fuentealba et al., 2015; Furutachi et al., 2015). V-SVZ NSCs are multipotent and are able to generate multiple neuron subtypes, astrocytes and oligodendrocytes (Menn et al., 2006; Mizrak et al., 2019; Obernier and Alvarez-Buylla, 2019). Most adult NSCs in the V-SVZ remain in an inactive and mitotically quiescent state, for long periods of time. A relatively small proportion of the NSCs in the V-SVZ enter the cell cycle at any one point in time and divide to generate more committed progeny. It has recently been described that most V-SVZ NSCs undergo symmetric, differentiating cell divisions but ~20% self-renew and remain in the V-SVZ for several months before they generate progeny (Obernier et al., 2018).

NSCs of the V-SVZ are polarized with an apical cilium that projects through the ependymal (E cells) lining of the ventricles into the cerebrospinal fluid (CSF) of the LV, and a long basal process that contacts blood vessels. The V-SVZ NSCs organize the E cells into pinwheel structures (Mirzadeh et al., 2008). During adult life, quiescent NSCs are activated to generate transient amplifying progenitors (C cells), which can divide further (three to four times) to give rise to neuroblasts (A cells) (Doetsch et al., 1999; Obernier et al., 2018). The newly formed neuroblasts in the V-SVZ migrate to the OB through the rostral migratory stream (RMS), where they differentiate into local interneurons that integrate into the local circuitry (Obernier and Alvarez-Buylla, 2019). V-SVZ NSCs are heterogeneous and, depending on their location, give-rise to different subtypes of interneurons of the OB (Merkle et al., 2007; Giachino et al., 2014; Giachino and Taylor, 2014; Merkle et al., 2014).

In addition to the NSCs in the lateral wall (LW) of the LV, recently gliogenic stem cells have been identified in the lateral septal wall (LSW) (Mizrak et al., 2019; Delgado et al., 2021), as well as a novel population of neurogenic NSCs in the dorsal septum (Lampada et al., 2022). LSW NSCs are predominantly in a quiescent state and induce pinwheel structures in the ependymal lining of the LV (Lampada et al., 2022). Stem cells in the LSW and V-SVZ have different lineage biases toward glia or neurons, indicating some mode of differential fate specification (Mizrak et al., 2019; Kjell et al., 2020; Delgado et al., 2021; Lampada et al., 2022). The roles of the stem cell populations in the forebrain outside the classic V-SVZ niche remain unclear.

## NSCs of the SGZ niche

Like the NSCs of the V-SVZ, the NSCs of the adult SGZ of the DG are formed during embryonic development. Their precursors acquire a radial morphology reminiscent of adult NSCs, localize to the SGZ of the DG and become quiescent during the early postnatal period (Berg et al., 2019). In contrast to V-SVZ NSCs that mostly divide symmetrically, NSCs in the SGZ predominantly divide asymmetrically to generate a daughter stem cell and an intermediate progenitor cell or an astrocyte (Bonaguidi et al., 2011). However, a fraction of SGZ NSCs do divide symmetrically to expand the stem cell pool (Bonaguidi et al., 2011; Obernier et al., 2018; Pilz et al., 2018).

DG NSCs predominantly generate glutamatergic granule neurons and to a less extent astrocytes but not oligodendrocytes (van Praag et al., 2002; Bonaguidi et al., 2011; Lugert et al., 2012; Rolando et al., 2016; Bonzano et al., 2018; Pilz et al., 2018; Obernier and Alvarez-Buylla, 2019). The adult SGZ contains morphologically discrete populations of NSCs (Lugert et al., 2010, 2012). Radial and horizontal NSCs (type-1 cells) in the adult SGZ give-rise to mitotic intermediate progenitor cells (IPs; type-2 cells). IPs (type-2 cells) are subdivided into early IPs (type-2a cells) and late IPs (type-2b cells) (Lugert et al., 2012). SGZ NSCs generate early IPs (type-2a cells) through asymmetric divisions. Early IPs (type-2a cells) self-replicate through symmetric divisions and then generate late IPs (type-2b cells). Late IPs (type-2b) give-rise to neuroblasts (type-3 cells), which is a pool of committed progenitors that eventually differentiate into mature granule cells of the DG (Lugert et al., 2012; Goncalves et al., 2016; Engler et al., 2018b; Obernier and Alvarez-Buylla, 2019).

## Notch signaling pathway overview

**Figure 1:**
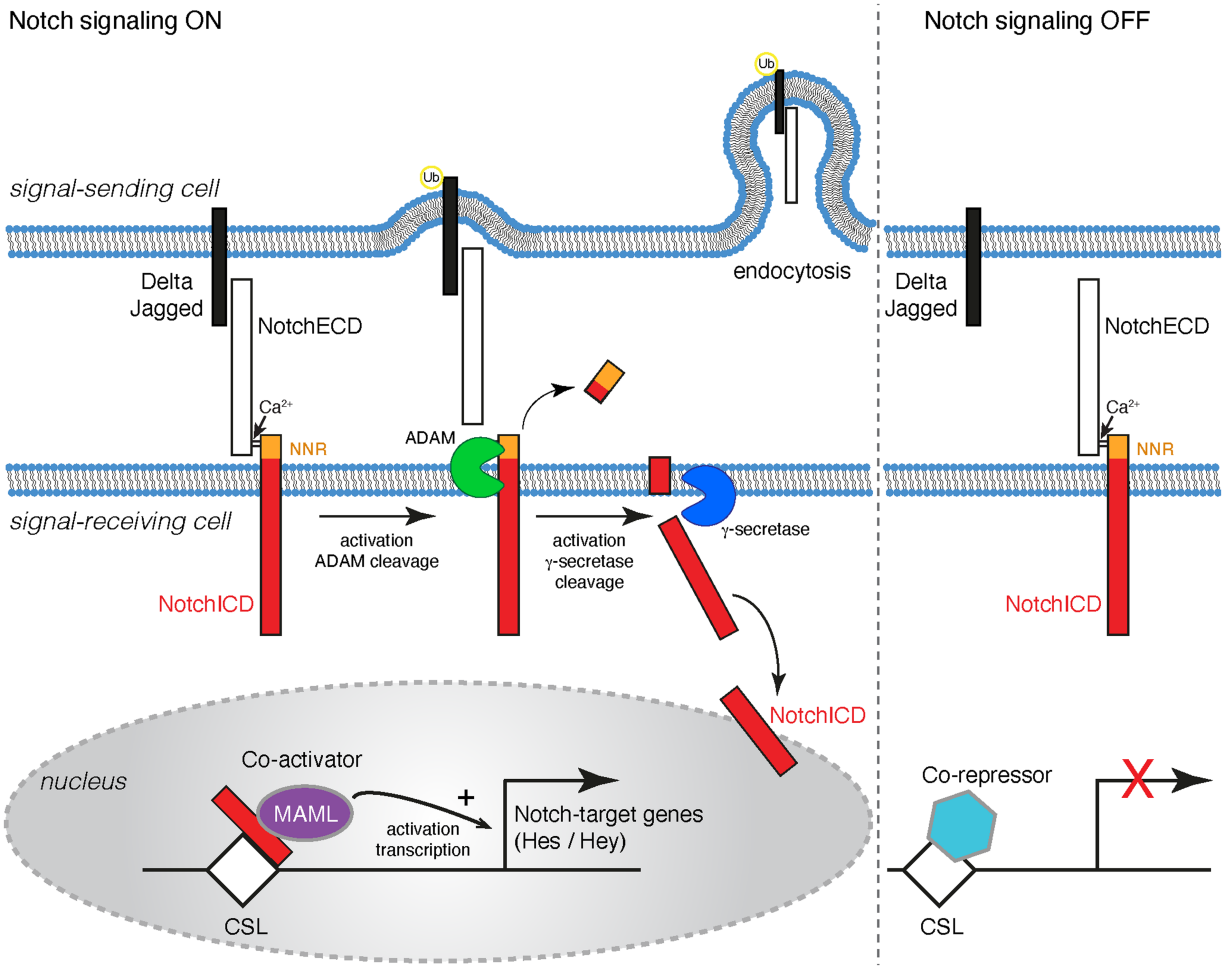
Schematic representation of the Notch signaling cascade. Notchs and their cognate ligands (Deltas and Jaggeds) are transmembrane proteins. Notch signaling occurs between cells that are in contact with one another. Notch signaling is activated when a ligand on a signal-sending cell binds to the Notch extracellular domain (NotchECD) on a signal-receiving cell. The Notch/ligand interaction triggers the ubiquitination (Ub) of the ligand intracellular domain, which results in endocytosis into the signal-sending cell. Endocytosis generates a pulling force that causes a conformational change on the NotchECD and reveals a proteolytic cleavage site for ADAM10/17 in the negative regulatory region (NRR) of the receptor. Two consecutive proteolytic cleavage events on the transmembrane portion of the Notch receptor are initiated. The first cleavage event by ADAM and the second by γ-secretase. These cleavage events release the Notch intracellular domain (NotchICD) into the cytoplasm of the signal-receiving cell. The The NotchICD translocates translocate to the nucleus where it interacts with the DNA-binding protein CSL and the co-activator Mastermind (MAML) to promote target gene transcription. Some Notch targets include genes of the *Hes* and *Hey* families. When Notch signaling is OFF, Notch receptors at the cell surface are heterodimers where the ADAM cleavage site is occluded. CSL is in the nucleus and interacts with co-repressors to inhibit the transcription of target genes.

The mammalian genome contains four Notch receptor (*Notch1-4*) and five canonical ligand (*Dll1, Dll3 and Dll4* and *Jag1 and Jag2*) genes. Both the receptors and their ligands are type I transmembrane proteins, presented on the surface of cells and thus enabling direct cell-to-cell communication (Figure 1). The receptors and the ligands both contain EGF repeats in their ectodomains as well as specialized domains for ligand-receptor interaction and activation. The ligands have relatively short intracellular domains which are important for surface presentation and activation of the Notchs by promoting endocytosis and mechanical conformational changes in the receptor extracellular domains (Figure 1; Giaimo and Borggrefe, 2018; Zhang et al., 2018; Sprinzak and Blacklow, 2021).

Notch receptors are synthesized in the endoplasmic reticulum (ER) as proproteins and are then proteolytically processed by a Furin-like protease and post-translationally modified to generate the mature heterodimeric receptor. The mature Notch receptors are expressed on the plasma membrane with the Notch extracellular domain (NECD) linked to the transmembrane-intracellular domain through a calcium bridge (Figure 1; Giaimo and Borggrefe, 2018; Zhang et al., 2018; Sprinzak and Blacklow, 2021).

Binding of a Notch to a ligand triggers the ubiquitination of the ligand, which results in endocytosis of the ligand and the bound NECD into the signal-sending cell. An endocytosis-mediated conformational change on the NECD reveals a proteolytic cleavage site for ADAM10/17 in the negative regulatory region (NRR) of the Notch receptor which facilitates the removal of the NECD. In the absence of a Notch/ligand interaction, the LNR (Lin-12/Notch Repeat) domains within the NRR prevent access and cleavage by ADAM proteases at the NECD, maintaining the Notch receptor in an inactive conformation (Sanchez-Irizarry et al., 2004; Gordon et al., 2007, 2009; Weinmaster and Fischer, 2011; Meloty-Kapella et al., 2012; Musse et al., 2012; Giaimo and Borggrefe, 2018; Handford et al., 2018; Lovendahl et al., 2018; Salazar and Yamamoto, 2018; Sprinzak and Blacklow, 2021). Cleavage by ADAM prevents reassociation of the ectodomain and results in intramembrane proteolysis of the remaining Notch transmembrane protein fragment by γ-secretase to release the intracellular domain (ICD) of the receptor. Apart from being present at the cell membrane, γ-secretase is also present in intracellular membrane compartments, including endosomes and lysosomes. It has been documented that γ-secretase can cleave Notch at the plasma membrane as well as in endocytic compartments, however it remains unclear whether γ-secretase primarily processes Notch receptors at the plasma membrane, within endocytic vesicles following Notch endocytosis, or in both compartments (Gupta-Rossi et al., 2004; Chyung et al., 2005; Hansson et al., 2005; Lange et al., 2011; Salazar and Yamamoto, 2018; Schnute et al., 2018; Steinbuck and Winandy, 2018). The intracellular domain of Notch proteins acts as a transcriptional regulator by binding to the CSL DNA-binding protein (RBPj, CBF1, Suppressor of Hairless, Lag-1), recruiting transcriptional coactivators and epigenetic regulators to target genes (Figure 1; Bray, 2016; Giaimo and Borggrefe, 2018; Lovendahl et al., 2018; Oswald and Kovall, 2018; Falo-Sanjuan and Bray, 2020; Sprinzak and Blacklow, 2021).

The NICD-CSL-MAML trimeric complex then recruits other co-activators and histone acetyltransferases to promote transcriptional activation of target genes. In the absence of Notch signal activation, CSL remains in the nucleus and interacts with several co-repressors to inhibit target gene transcription (Bray, 2016; Giaimo and Borggrefe, 2018; Lovendahl et al., 2018; Oswald and Kovall, 2018; Falo-Sanjuan and Bray, 2020; Sprinzak and Blacklow, 2021). The best characterized Notch targets genes include members of the hairy and enhancer of split (*Hes*) and hairy and enhancer of split-related with YRPW motif (*Hey*) families (Figure 1). HES and HEY proteins in the brain control the expression of several proneural genes (*Ascl1*, *Atoh1*, *Neurog1* and *Neurog2*) and are therefore important for NSC cell fate determination and neuronal differentiation (Bigas and Porcheri, 2018; Engler et al., 2018b; Urban et al., 2019).

Notch signaling is an important cell fate regulator in different organs, including in the brain, from embryogenesis through to adult homeostasis (Bray, 2016; Ho et al., 2018; Engler et al., 2018b; Ho et al., 2020). Control of NSC activity and fate is crucial for brain development and homeostasis. Misregulation of the Notch pathway in the central nervous system has been implicated in various pathological conditions, from progressive neurodegenerative diseases to cancer (Giachino et al., 2015; Zhang et al., 2018; Ho et al., 2020; Parmigiani et al., 2020, 2022). In this review, we will mainly discuss the regulation and function of the Notch signaling pathway in adult neurogenesis under homeostatic conditions with a focus on rodents and genetic experiments.

## Notch signaling in adult neurogenesis

**Figure 2:**
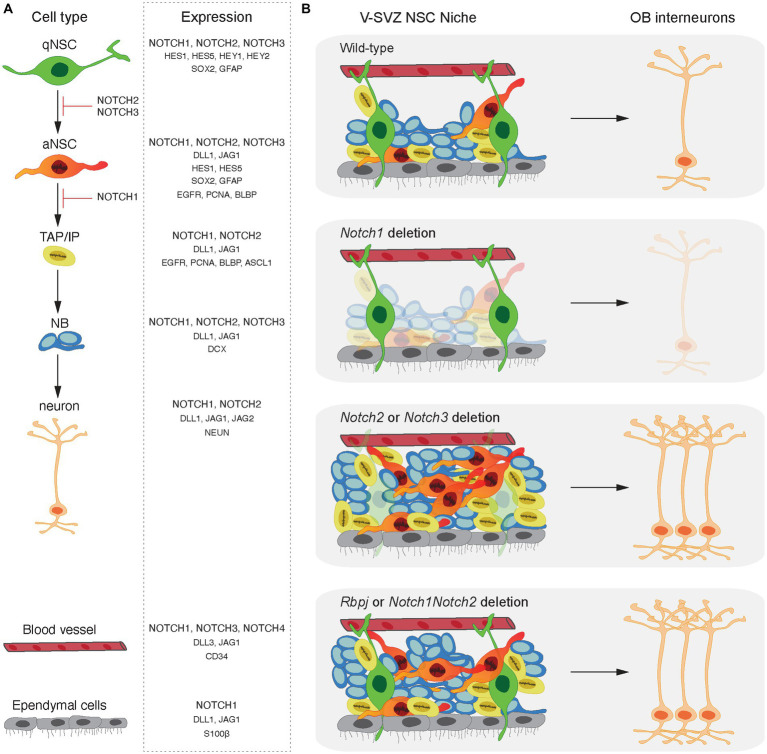
Notch signaling in adult neurogenesis. **(A)** Expression patterns of Notch ligands, receptors and downstream effectors in the neuronal lineage and cells of the adult NSC niche. Expression of Notch receptor paralogues and Rbpj has been documented in all cell types of the mouse adult-born neuronal lineage from NSCs to neurons. However, downstream effectors of the Notch signaling cascade (Hes and Hey factors) are present only in NSCs in both quiescent and activated states. Dll and Jagged ligands are expressed throughout the lineage apart from quiescent NSCs. **(B)** Functions of the Notch receptors and their common downstream effector, Rbpj, in the V-SVZ. Notch1 plays a role on the maintenance of the activated NSCs whereas Notch2 and Notch3 maintain NSC quiescence in the adult V-SVZ. Rbpj deletion and concomitant *Notch1Notch2* deletion result in similar phenotypes highlighting that Rbpj-mediated Notch signaling regulates NSC maintenance downstream of both Notch1 and Notch2 receptors.

Expression of all four Notch receptor paralogs has been documented in various cell types of the adult mouse brain including by NSCs (Notch1, Notch2 and Notch3), astrocytes (Notch1 and Notch2), neurons (Notch1 and Notch2), endothelial cells (Notch1 and Notch4) and vascular smooth muscle cells and pericytes (Notch3) (Basak et al., 2012; Ehret et al., 2015; Llorens-Bobadilla et al., 2015; Shin et al., 2015; Kawai et al., 2017; Rieskamp et al., 2018; Engler et al., 2018a; Zhang et al., 2019; Ho et al., 2020). Furthermore, Notch receptors and many of their ligands are expressed by NSCs, progenitors and neuroblasts throughout the adult neurogenic lineage. However, downstream targets of the Notch pathway (Hes and Hey transcription factors) are only expressed by NSCs. This indicates that Notch signaling in the neurogenic niches is highly regulated and not only due to receptor or ligand expression by a cell (Figure 2A; Stump et al., 2002; Irvin et al., 2004; Nyfeler et al., 2005; Carlen et al., 2009; Aguirre et al., 2010; Lugert et al., 2010; Basak et al., 2012; Kawaguchi et al., 2013; Giachino et al., 2014; Lavado and Oliver, 2014; Ehret et al., 2015; Kawai et al., 2017; Semerci et al., 2017; Engler et al., 2018a,b; Zhang et al., 2018; Sueda et al., 2019; Zhang et al., 2019; Harada et al., 2021). Notch signaling can be regulated at multiple levels modulating the strength and dynamics of the signal outcome. The expression patterns of different Notch receptors and ligands, different modifications affecting ligand-receptor interactions and factors affecting the transcriptional activity of the Notch pathway contribute to differential regulation of NSC behavior and consequently adult neurogenesis.

## Differential cell autonomous functions of Notch receptors on NSCs

Loss of function experimental approaches manipulating the expression of different components of the Notch pathway by NSCs combined with lineage tracing of the NSC population and their progeny have uncovered crucial roles for Notch signaling in adult mouse neurogenesis. Early studies investigated a cell autonomous role of Notch signaling in neurogenesis by ablating *Rbpj*, the common downstream effector of all Notch receptors. *Rbpj* deletion in NSCs of the V-SVZ and SGZ of the adult mouse brain caused an initial transient increase in neurogenesis that is followed by a total depletion of the NSC pool and loss of neurogenesis in the long-term (Ehm et al., 2010; Imayoshi et al., 2010; Lugert et al., 2010). These data indicated that Notch signaling plays a pivotal role in the regulation of NSC maintenance in both V-SVZ and SGZ neurogenic niches. However, any Rbpj-dependent but Notch-independent effects on adult neurogenesis could not be excluded by these experiments, and therefore further studies investigated the precise role of individual Notch receptors during adult neurogenesis.

The Notch1 receptor was found to be an important mediator of maintenance of a pool of proliferating undifferentiated cells in the adult SGZ and conditional deletion of *Notch1* from NESTIN positive progenitors led to a decrease in NSCs, IPs, NBs and mature neurons (Ables et al., 2010). Similarly, in the V-SVZ, ablation of *Notch1* from NESTIN positive progenitors resulted in a persistent defect in adult neurogenesis accompanied by a loss of mitotic progenitors and NBs. Interestingly, it was shown that Notch1 promotes the maintenance of aNSCs. Genetic ablation of *Notch1* from NSCs led to selective loss of the aNSC pool while qNSCs remain unchanged (Basak et al., 2012). qNSCs induced to enter cell cycle during regeneration and aging became Notch1-dependent and consequently failed to fully reinstate neurogenesis following *Notch1* deletion (Basak et al., 2012). This indicates that the qNSCs and aNSCs are in the same linage and that Notch1 is required at the activated stage (Basak et al., 2012). The observation that conditional deletion of *Rbpj* or *Notch1* from NSCs of the V-SVZ causes partially different phenotypes left open the possibility that other Notch receptors could functionally compensate the signals that repress activation of qNSCs through Rbpj.

Single cell transcriptomic analysis of NSCs in the adult V-SVZ reveals that *Notch2* expression is enriched in qNSCs (Llorens-Bobadilla et al., 2015). Engler et al. (2018a), performed conditional *Notch2* deletion in NSCs and revealed that Notch2 regulates adult NSC quiescence in the V-SVZ. Combinatorial conditional genetic deletion of *Notch1* and *Notch2* and *Rbpj* in NSCs uncovered distinct functions of Notch1 and Notch2 in regulating neurogenesis (Engler et al., 2018a).

Genome-wide gene expression analysis showed that *Notch2* deletion from V-SVZ NSCs affected genes associated with NSC proliferation and differentiation (Engler et al., 2018a). Indeed, qNSCs were rapidly activated and entered cell cycle shortly after deletion of *Notch2*, *Notch1Notch2* or *Rbpj*, but were not affected by *Notch1* deletion. The loss of *Notch2* led to an initial precocious differentiation and increased neurogenesis in the V-SVZ resulting in more neurons in the OB. However, 300 days after *Notch2* deletion the NSC pool had been exhausted leading to reduced V-SVZ neurogenic capacity, and a premature aging-like phenotype. Importantly, concomitant *Notch1Notch2* and *Rbpj* deletion gave similar phenotypes highlighting that Rbpj-mediated Notch signaling regulates NSC maintenance downstream of both Notch1 and Notch2 (Engler et al., 2018a).

Similarly, *Notch2* deletion in Hes5 positive SGZ NSCs induced a rapid loss of qNSCs and an increase in proliferative progenitors, as well as NBs (Zhang et al., 2019). The effects culminated in a premature exhaustion of the SGZ NSC pool and neurogenic decline by 100 days after gene inactivation (Zhang et al., 2019). RNA-sequencing revealed that *Notch2* deletion downregulated quiescent NSC-associated genes while genes associated with NSC activation were upregulated (Zhang et al., 2019). *Notch2* activation by expression of the active form of *Notch2* (*Notch2ICD*) in SGZ NSCs maintained qNSCs, blocked NSC entry into cell cycle, and decreased mitotic progenitor, NB and neuron production (Zhang et al., 2019). Single cell RNA-sequencing of adult SGZ NSCs and their progeny indicate that quiescent adult NSCs express genes associated with different signaling pathways in the niche (Shin et al., 2015). Genes related by Notch signaling, including *Notch2*, are downregulated upon NSC exit from quiescence and entry into mitosis, further supporting the role of Notch2 in the regulation of NSC quiescence (Shin et al., 2015). Recently, a population of quiescent NSCs with latent neurogenic potential was identified in the dorsal LSW of adult mice (Lampada et al., 2022). These dorsal LSW NSCs are dependent on Notch2 signaling which regulates quiescence and prevents their entry into the cell cycle and neurogenesis. These LSW NSCs respond to acute stress and elevated serotonin levels (Lampada et al., 2022). *Notch2* deletion increases proliferation and NB production in the dorsal LSW and generates new GABAergic interneurons that integrate into septal nuclei but which do not migrate to the OB (Lampada et al., 2022).

Similar to Notch2, Notch3 has also been described to play an important role in the maintenance of quiescent NSCs in the adult V-SVZ. Notch3 is expressed by qNSCs located at the lateral and ventral walls of the V-SVZ (Kawai et al., 2017). Germline deletion of *Notch3* led to a decrease in qNSCs, TAPs, NBs and Calbindin positive OB neurons without affecting the pool of aNSCs. It was suggested that qNSCs in *Notch3* knockout mice increase their activation but fail to complete lineage progression (Kawai et al., 2017). Nevertheless, acute knockdown of *Notch3* in NSCs in the lateral wall of the adult V-SVZ promotes qNSCs activation (Kawai et al., 2017; Rieskamp et al., 2018). Notch3 has also been associated with qNSCs in the adult SGZ. Using a transgenic mouse model overexpressing *Notch3*, Notch3 expression reduced precursor cell activation and proliferation without affecting levels of neurogenesis in the adult DG (Ehret et al., 2015).

Collectively, a differential mode of function of three Notch receptors expressed by NSCs has unveiled a pleiotropy in Notch signal function *in vivo*. Interestingly, the functions of the different Notch receptors are not compensated in NSCs. While Notch1 plays a role in the maintenance of aNSCs, Notch2 and Notch3 maintain NSC quiescence in both the adult V-SVZ and SGZ (Figure 2B; Ables et al., 2010; Ehm et al., 2010; Imayoshi et al., 2010; Basak et al., 2012; Ehret et al., 2015; Kawai et al., 2017; Engler et al., 2018a; Zhang et al., 2019). Even though the distinct functions of the Notch receptors can be attributed, at least in part, to their differential expression patterns on the different types of cells in the adult neurogenic lineages, an overlap in Notch1, Notch2 and Notch3 receptor expression by NSCs is clear (Ehret et al., 2015; Kawai et al., 2017; Engler et al., 2018a). The precise mechanism underlying the differential mode of function of Notch receptors and how they control different aspects of NSCs activity remains elusive.

## Notch-mediated transcriptional responses in NSCs

Several transcriptional targets of the Notch pathway have been directly implicated in the maintenance and activation of quiescent NSCs and in their subsequent neuronal differentiation. *Hes5* expression distinguishes NSCs from intermediate progenitors in the neurogenic niches of the adult brain. However, both quiescent and activated NSCs in the V-SVZ and SGZ express Hes5 (Lugert et al., 2010, 2012; Giachino et al., 2014). The dynamics of Hes factor expression have been shown to control the balance between quiescent and activated states of NSCs during neurodevelopment. Specifically, Hes1/Hes5 levels oscillate out-of-phase with *Ascl1*, thereby promoting proliferation of mouse embryonic NSCs (Imayoshi et al., 2013). It remains unclear whether Hes factors are sustained or oscillatory in adult NSCs *in vivo*, however, *Hes1* expression does oscillate in some cells in cultured slices of the neurogenic regions of the adult brain (Sueda et al., 2019). Higher levels of *Hes1* expression were observed in qNSCs compared to aNSCs in both the V-SVZ and SGZ (Sueda et al., 2019; Sueda and Kageyama, 2020). Conditional deletion of *Hes1* in NSCs of *Hes3*/*Hes5*/*Hey1* knockout mice resulted in an activation of *Ascl1* expression and a transient increase in neurogenesis. This eventually resulted in depletion of the NSCs and termination of neurogenesis. Conversely, sustained *Hes1* expression repressed *Ascl1* expression and inhibited neurogenesis (Sueda et al., 2019; Sueda and Kageyama, 2020). Recently it has been suggested that the Notch-HEY1 axis also plays an important role in adult NSC ontogeny and long-term maintenance of quiescent embryonic NSCs through to adulthood. Interestingly, Hey1 has a non-oscillatory expression in NSCs (Harada et al., 2021).

The quiescence of adult NSCs has been linked to the expression of inhibitor of DNA-binding factors (IDs) in both the V-SVZ and SGZ (Llorens-Bobadilla et al., 2015; Shin et al., 2015; Blomfield et al., 2019; Zhang et al., 2019). In mammals, the *Id* family consists of four genes, *Id1-4*, that have been linked with stemness and proliferation (Niola et al., 2012). *Id2* and *Id3* expression are associated with quiescence in the adult V-SVZ (Llorens-Bobadilla et al., 2015). *Id3* and *Id4* were shown to be expressed by qNSCs in the DG and were both downregulated by mitotic cells (Shin et al., 2015). The ID1, ID3 and ID4 proteins are expressed in the SGZ of the DG of adult mice, and ID4 is expressed by the majority of the RG, and primarily by qNSCs (Blomfield et al., 2019). ID factors interact directly with Hes1 to form heterodimers. These heterodimers are responsible for releasing the negative feedback autorepression of Hes1 on its own promoter, in this way retaining *Hes1* expression and NSC maintenance (Bai et al., 2007). By combining mathematical modeling with analysis of published single-cell transcriptomic data, it was found that Notch signaling and ID factors control neurogenesis in a complementary manner. ID expression maintains NSCs in a quiescence state by blocking proneural gene expression and activity. Downregulation of IDs releases the complete repression of proneural activity mediated by sustained high levels of Hes expression. This promotes cell cycle entry by enabling proneural factor expression (Boareto et al., 2017). Recently, it was uncovered that quiescence in the adult SGZ is maintained by the expression of ID4 downstream of Notch2 signaling. Gene expression analysis and chromatin immunoprecipitation experiments identified *Id4* as a direct target of Notch2 signaling. *Id4* knockdown partially rescues the proliferation defect induced by *Notch2ICD* overexpression in SGZ NSCs, indicating that Notch2 and ID4 control proliferation in a complementary fashion *in vitro*. Genetic deletion and overexpression of *Id4* in adult SGZ NSCs confirmed that ID4 maintains NSC quiescence *in vivo* (Zhang et al., 2019).

## Ligand-mediated regulation of Notch signaling in NSCs

The activation of Notch receptors on adult NSCs requires their interaction with cells expressing a ligand, underlying the importance of the niche microenvironment in Notch signal regulation (Figure 1). Dll1 and Jagged1 were found to control Notch signaling in the V-SVZ and SGZ of adult mice (Nyfeler et al., 2005; Kawaguchi et al., 2013; Lavado and Oliver, 2014; Semerci et al., 2017). Apart from activating Notch on neighboring cells (*trans* interaction) Dll ligands are able to inactivate Notch signaling within the same cell (*cis* interaction/inhibition) (Sprinzak et al., 2010). In the adult V-SVZ, Dll1 was suggested to play an essential role in the maintenance of qNSCs (Kawaguchi et al., 2013). *Dll1* deletion from V-SVZ NSCs decreased the number of qNSCs while increasing the number of aNSCs, TAPs and NBs. Dll1 is expressed by cells negative for NICD expression that resided in close proximity to quiescent V-SVZ NSCs. Therefore, a model whereby Dll-expressing aNSCs control the dormancy of quiescent NSCs has been proposed (Kawaguchi et al., 2013). Jagged1 expression has been detected in both the neurogenic V-SVZ and DG regions of the adult brain (Stump et al., 2002; Irvin et al., 2004). Jagged1 was found to be expressed in the V-SVZ in a mutually exclusive manner to Notch1. Mice double hemizygous null for *Jagged1* and *Notch1* show reduced mitosis in the V-SVZ (Nyfeler et al., 2005). Ablation of *Jagged1* from adult V-SVZ neurospheres blocks NSC self-renewal potential, but do not affect their differentiation potential *in vitro* (Nyfeler et al., 2005). Conversely, treatment of V-SVZ neurospheres with a dimeric soluble Jagged1 induces NSC self-renewal and promotes neurogenic capacity *in vitro*. These findings suggest that Jagged1 plays a role in the maintenance of NSCs in the V-SVZ (Nyfeler et al., 2005). Similar to the V-SVZ, Jagged1 was found to play an essential role in neurogenesis in the adult SGZ by regulating NSC maintenance and proliferation. Conditional genetic deletion of *Jagged1* from SGZ NESTIN positive progenitors caused a transient increase in neurogenesis. However, *Jagged1* deletion eventually led to depletion of the SGZ NSC pool and obstructed neurogenesis (Lavado and Oliver, 2014).

It has been shown that Fringe-modified Notch receptors respond differently to ligand activating signals and modulate Notch activity. Fringe proteins (Lunatic, Manic and Radical in mammals) are β3-N-acetylglucosaminyltransferases that modify O-fucose on epidermal growth factor-like (EGF) repeats in the NECD. Specific fringe modifications therefore can enhance Notch binding to Dll1 resulting in activation of Notch signaling whereas others inhibit Notch activation by Jagged1 (Bray, 2016; Kakuda and Haltiwanger, 2017; Zhang et al., 2018). Recently, it has been described that Lunatic fringe (LFNG) is selectively expressed by NSCs in the adult SGZ together with Notch1. Additionally, Jagged1 was found to be expressed by amplifying neural progenitor cells and Dll1 by granule neurons of the DG, in close proximity to the NSCs. LFNG-mediated Notch signaling was therefore suggested to control SGZ NSC maintenance in the adult brain since genetic deletion of *Lfng* from NSCs leads to increased NSC proliferation, reduced numbers of amplifying neural progenitors and an increased tendency for astrocytic differentiation. Similar to *Lfng*, *Dll1* deletion leads to an increase in mitotic NSCs and less amplifying neural progenitors. On the other hand, genetic deletion of *Jagged1* causes NSCs to re-enter the cell cycle leading to an increase in amplifying neural progenitor production (Semerci et al., 2017). In summary, Notch signaling and its ligands play pivotal roles as niche molecules in the control of NSC activity and neurogenesis in the V-SVZ and SGZ of the DG. How the same pathway activated by different receptors can have such distinct functions in the different cells of the neurogenic lineages remains to be determined.

## Discussion

In this review, we present an overview of our current knowledge about how the Notch signaling controls adult neurogenesis, with emphasis given to the roles of the pathway in NSC activity. It is now widely accepted that Notch signaling plays a pivotal role on NSC maintenance and neurogenesis. Different Notch receptor paralogues are able to control either the quiescent or activated state of NSCs through Rbpj and transcriptional regulation of target genes. In addition to Notch receptors, several Notch ligands and downstream transcriptional effectors play key roles in NSC maintenance and neurogenesis. However, the precise molecular mechanism that differentially controls Notch signaling in NSCs remains elusive and needs further investigation. The pleiotropic functions of Notch signaling in adult neurogenesis could be attributed to the complex nature and regulation of the pathway. Differential ligand-mediated activation of Notch receptors might control the quiescent or activated NSC state and maintenance. Additionally, differential transcriptional responses downstream of the Notch receptors might control the activation state of NSCs. It is intriguing to speculate that different Notch receptors modulate distinct gene networks and/or overlapping gene networks with distinctive strengths and dynamics. Additionally, the architecture of the NSC niches certainly adds another level of complexity to the regulation of Notch signaling in NSCs. Notch signaling mediates cell-to-cell communication between neighboring cells, and niche cells control NSC behavior, it could be that the expression of Notch ligands and/or other Notch interactors within the niche affects NSC fate and maintenance. The identification of new upstream or downstream effectors of the Notch signaling pathway will pave the way to a better understanding of NSC biology and fate switch.

There are still a number of important open questions about the role of Notch signaling in adult neurogenesis that need to be addressed in the future. Many of these unknowns are manifested in the fact that our current knowledge of Notch signaling cannot explain why cells in the same neurogenic niche express multiple Notch receptors which regulate either none compensated different processes or exert different functions.

Although it is clear that different Notch receptors play different roles in regulating the activity and fate of NSCs, how this is achieved for receptors that interact with the same set of ligands and use the same DNA binding molecule (CSL protein) is still not known. For example, it still remains unclear whether Notch receptors (Notch1-3) compete with each other for ligands, downstream effector proteins or target genes in order to modulate the response of the cell to different Notch receptor signals. Additionally, it is not known whether different ligands can activate Notch receptors to induce different downstream effectors of the pathway within the same cell. Although Notch1 has been shown to be target of its own signal in some cells, it remains to be shown whether Notch receptors can cross regulate the expression of other Notch receptors at the transcriptional level in adult NSCs. Finally, the discrete or compensatory roles of the individual Notch ligands in the adult neurogenesis remain to be elucidated. In summary, there remains much to be learnt about the Notch signaling in the control of NSCs in the adult brain, and future findings may have important implications for other organs and even in diseases including cancer.

## Author contributions

AL and VT wrote and edited the manuscript and designed and generated the figures. All authors contributed to the article and approved the submitted version.

## Funding

This work was supported by the Swiss National Science Foundation (31003A_162609 and 31003A_182388 to VT).

## Conflict of interest

The authors declare that the research was conducted in the absence of any commercial or financial relationships that could be construed as a potential conflict of interest.

## Publisher’s note

All claims expressed in this article are solely those of the authors and do not necessarily represent those of their affiliated organizations, or those of the publisher, the editors and the reviewers. Any product that may be evaluated in this article, or claim that may be made by its manufacturer, is not guaranteed or endorsed by the publisher.

## References

[ref1] Ables J. L. Decarolis N. A. Johnson M. A. Rivera P. D. Gao Z. L. Cooper D. C.. (2010). Notch1 is required for maintenance of the reservoir of adult hippocampal stem cells. J. Neurosci. 30, 10484–10492. doi: 10.1523/JNEUROSCI.4721-09.2010, PMID: 20685991 PMC2935844

[ref2] Aguirre A. Rubio M. E. Gallo V. (2010). Notch and EGFR pathway interaction regulates neural stem cell number and self-renewal. Nature 467, 323–327. doi: 10.1038/nature09347 20844536 PMC2941915

[ref3] Altman J. (1962). Are new neurons formed in brains of adult mammals. Science 135, 1127–1128. doi: 10.1126/science.135.3509.1127 13860748

[ref4] Altman J. Das G. D. (1965). Autoradiographic and histological evidence of postnatal hippocampal neurogenesis in rats. J. Comp. Neurol. 124, 319–335. doi: 10.1002/cne.901240303, PMID: 5861717

[ref5] Bai G. Sheng N. Y. Xie Z. H. Bian W. Yokota Y. Benezra R.. (2007). Id sustains Hes1 expression to inhibit precocious neurogenesis by releasing negative autoregulation of Hes1. Dev. Cell 13, 283–297. doi: 10.1016/j.devcel.2007.05.014, PMID: 17681138

[ref6] Basak O. Giachino C. Fiorini E. Macdonald H. R. Taylor V. (2012). Neurogenic subventricular zone stem/progenitor cells are Notch1-dependent in their active but not quiescent state. J. Neurosci. 32, 5654–5666. doi: 10.1523/JNEUROSCI.0455-12.2012, PMID: 22514327 PMC6703480

[ref7] Basak O. Krieger T. G. Muraro M. J. Wiebrands K. Stange D. E. Frias-Aldeguer J.. (2018). Troy+ brain stem cells cycle through quiescence and regulate their number by sensing niche occupancy. Proc. Natl. Acad. Sci. U. S. A. 115, E610–E619. doi: 10.1073/pnas.1715911114 29311336 PMC5789932

[ref8] Berg D. A. Belnoue L. Song H. J. Simon A. (2013). Neurotransmitter-mediated control of neurogenesis in the adult vertebrate brain. Development 140, 2548–2561. doi: 10.1242/dev.088005, PMID: 23715548 PMC3666382

[ref9] Berg D. A. Su Y. J. Jimenez-Cyrus D. Patel A. Huang N. Morizet D.. (2019). A common embryonic origin of stem cells drives developmental and adult neurogenesis. Cells 177, 654–668.e15. doi: 10.1016/j.cell.2019.02.010, PMID: 30929900 PMC6496946

[ref10] Bigas A. Porcheri C. (2018). Notch and stem cells. Adv. Exp. Med. Biol. 1066, 235–263. doi: 10.1007/978-3-319-89512-3_12 30030830

[ref11] Blomfield I. M. Rocamonde B. Masdeu M. D. Mulugeta E. Vaga S. Van Den Berg D. L. C.. (2019). Id4 promotes the elimination of the pro-activation factor Ascl1 to maintain quiescence of adult hippocampal stem cells. elife 8:e48561. doi: 10.7554/eLife.48561, PMID: 31552825 PMC6805120

[ref12] Boareto M. Iber D. Taylor V. (2017). Differential interactions between Notch and ID factors control neurogenesis by modulating Hes factor autoregulation. Development 144, 3465–3474. doi: 10.1242/dev.152520, PMID: 28974640 PMC5665482

[ref13] Boldrini M. Fulmore C. A. Tartt A. N. Simeon L. R. Pavlova I. Poposka V.. (2018). Human hippocampal neurogenesis persists throughout aging. Cell Stem Cell 22:589. doi: 10.1016/j.stem.2018.03.015, PMID: 29625071 PMC5957089

[ref14] Bonaguidi M. A. Wheeler M. A. Shapiro J. S. Stadel R. P. Sun G. J. Ming G. L.. (2011). In vivo clonal analysis reveals self-renewing and multipotent adult neural stem cell characteristics. Cells 145, 1142–1155. doi: 10.1016/j.cell.2011.05.024, PMID: 21664664 PMC3124562

[ref15] Bonzano S. Crisci I. Podlesny-Drabiniok A. Rolando C. Krezel W. Studer M.. (2018). Neuron-astroglia cell fate decision in the adult mouse hippocampal neurogenic niche is cell-intrinsically controlled by COUP-TFI in vivo. Cell Rep. 24, 329–341. doi: 10.1016/j.celrep.2018.06.044, PMID: 29996095

[ref16] Bray S. J. (2016). Notch signalling in context. Nat. Rev. Mol. Cell Biol. 17, 722–735. doi: 10.1038/nrm.2016.94 27507209

[ref17] Calzolari F. Michel J. Baumgart E. V. Theis F. Gotz M. Ninkovic J. (2015). Fast clonal expansion and limited neural stem cell self-renewal in the adult subependymal zone. Nat. Neurosci. 18, 490–492. doi: 10.1038/nn.3963, PMID: 25730673

[ref18] Carlen M. Meletis K. Goritz C. Darsalia V. Evergren E. Tanigaki K.. (2009). Forebrain ependymal cells are Notch-dependent and generate neuroblasts and astrocytes after stroke. Nat. Neurosci. 12, 259–267. doi: 10.1038/nn.2268, PMID: 19234458

[ref19] Chyung J. H. Raper D. M. Selkoe D. J. (2005). Gamma-secretase exists on the plasma membrane as an intact complex that accepts substrates and effects intramembrane cleavage. J. Biol. Chem. 280, 4383–4392. doi: 10.1074/jbc.M409272200, PMID: 15569674

[ref20] Delgado A. C. Maldonado-Soto A. R. Silva-Vargas V. Mizrak D. Von Kanel T. Tan K. R.. (2021). Release of stem cells from quiescence reveals gliogenic domains in the adult mouse brain. Science 372:1205. doi: 10.1126/science.abg8467, PMID: 34112692

[ref21] Doetsch F. Caille I. Lim D. A. Garcia-Verdugo J. M. Alvarez-Buylla A. (1999). Subventricular zone astrocytes are neural stem cells in the adult mammalian brain. Cells 97, 703–716. doi: 10.1016/S0092-8674(00)80783-7 10380923

[ref22] Ehm O. Goritz C. Covic M. Schaffner I. Schwarz T. J. Karaca E.. (2010). RBPJ kappa-dependent signaling is essential for long-term maintenance of neural stem cells in the adult hippocampus. J. Neurosci. 30, 13794–13807. doi: 10.1523/JNEUROSCI.1567-10.2010, PMID: 20943920 PMC6633732

[ref23] Ehret F. Vogler S. Pojar S. Elliott D. A. Bradke F. Steiner B.. (2015). Mouse model of CADASIL reveals novel insights into Notch3 function in adult hippocampal neurogenesis. Neurobiol. Dis. 75, 131–141. doi: 10.1016/j.nbd.2014.12.018 25555543

[ref24] Encinas J. M. Michurina T. V. Peunova N. Park J. H. Tordo J. Peterson D. A.. (2011). Division-coupled astrocytic differentiation and age-related depletion of neural stem cells in the adult hippocampus. Cell Stem Cell 8, 566–579. doi: 10.1016/j.stem.2011.03.010, PMID: 21549330 PMC3286186

[ref25] Engler A. Rolando C. Giachino C. Saotome I. Erni A. Brien C.. (2018a). Notch2 signaling maintains NSC quiescence in the murine ventricular-subventricular zone. Cell Rep. 22, 992–1002. doi: 10.1016/j.celrep.2017.12.094, PMID: 29386140

[ref26] Engler A. Zhang R. R. Taylor V. (2018b). Notch and neurogenesis. Adv. Exp. Med. Biol. 1066, 223–234. doi: 10.1007/978-3-319-89512-3_11 30030829

[ref27] Falo-Sanjuan J. Bray S. J. (2020). Decoding the Notch signal. Develop. Growth Differ. 62, 4–14. doi: 10.1111/dgd.12644, PMID: 31886523

[ref28] Fuentealba L. C. Rompani S. B. Parraguez J. I. Obernier K. Romero R. Cepko C. L.. (2015). Embryonic origin of postnatal neural stem cells. Cells 161, 1644–1655. doi: 10.1016/j.cell.2015.05.041, PMID: 26091041 PMC4475276

[ref29] Furutachi S. Miya H. Watanabe T. Kawai H. Yamasaki N. Harada Y.. (2015). Slowly dividing neural progenitors are an embryonic origin of adult neural stem cells. Nat. Neurosci. 18:657. doi: 10.1038/nn.3989, PMID: 25821910

[ref30] Giachino C. Basak O. Lugert S. Knuckles P. Obernier K. Fiorelli R.. (2014). Molecular diversity subdivides the adult forebrain neural stem cell population. Stem Cells 32, 70–84. doi: 10.1002/stem.1520, PMID: 23964022 PMC4259462

[ref31] Giachino C. Boulay J. L. Ivanek R. Alvarado A. Tostado C. Lugert S.. (2015). A tumor suppressor function for Notch signaling in forebrain tumor subtypes. Cancer Cell 28, 730–742. doi: 10.1016/j.ccell.2015.10.008, PMID: 26669487

[ref32] Giachino C. Taylor V. (2014). Notching up neural stem cell homogeneity in homeostasis and disease. Front. Neurosci. 8:32. doi: 10.3389/fnins.2014.00032, PMID: 24611040 PMC3933793

[ref33] Giaimo B. D. Borggrefe T. (2018). Introduction to molecular mechanisms in Notch signal transduction and disease pathogenesis. Adv. Exp. Med. Biol. 1066, 3–30. doi: 10.1007/978-3-319-89512-3_1, PMID: 30030819

[ref34] Goncalves J. T. Schafer S. T. Gage F. H. (2016). Adult neurogenesis in the hippocampus: from stem cells to behavior. Cells 167, 897–914. doi: 10.1016/j.cell.2016.10.021 27814520

[ref35] Gordon W. R. Roy M. Vardar-Ulu D. Garfinkel M. Mansour M. R. Aster J. C.. (2009). Structure of the Notch1-negative regulatory region: implications for normal activation and pathogenic signaling in T-ALL. Blood 113, 4381–4390. doi: 10.1182/blood-2008-08-174748, PMID: 19075186 PMC2676092

[ref36] Gordon W. R. Vardar-Ulu D. Histen G. Sanchez-Irizarry C. Aster J. C. Blacklow S. C. (2007). Structural basis for autoinhibition of Notch. Nat. Struct. Mol. Biol. 14, 295–300. doi: 10.1038/nsmb1227, PMID: 17401372

[ref37] Gupta-Rossi N. Six E. Lebail O. Logeat F. Chastagner P. Olry A.. (2004). Monoubiquitination and endocytosis direct gamma-secretase cleavage of activated Notch receptor. J. Cell Biol. 166, 73–83. doi: 10.1083/jcb.200310098, PMID: 15240571 PMC2172142

[ref38] Handford P. A. Korona B. Suckling R. Redfield C. Lea S. M. (2018). Structural insights into Notch receptor-ligand interactions. Adv. Exp. Med. Biol. 1066, 33–46. doi: 10.1007/978-3-319-89512-3_2, PMID: 30030820

[ref39] Hansson E. M. Stromberg K. Bergstedt S. Yu G. Naslund J. Lundkvist J.. (2005). Aph-1 interacts at the cell surface with proteins in the active gamma-secretase complex and membrane-tethered Notch. J. Neurochem. 92, 1010–1020. doi: 10.1111/j.1471-4159.2004.02926.x, PMID: 15715652

[ref40] Harada Y. Yamada M. Imayoshi I. Kageyama R. Suzuki Y. Kuniya T.. (2021). Cell cycle arrest determines adult neural stem cell ontogeny by an embryonic Notch-nonoscillatory Hey1 module. Nat. Commun. 12:6562. doi: 10.1038/s41467-021-26605-0, PMID: 34772946 PMC8589987

[ref41] Ho D. M. Artavanis-Tsakonas S. Louvi A. (2020). The Notch pathway in CNS homeostasis and neurodegeneration. Wiley Interdiscipl. Rev. Develop. Biol. 9:e358. doi: 10.1002/wdev.358, PMID: 31502763

[ref42] Ho D. M. Guruharsha K. G. Artavanis-Tsakonas S. (2018). The Notch interactome: complexity in signaling circuitry. Adv. Exp. Med. Biol. 1066, 125–140. doi: 10.1007/978-3-319-89512-3_7, PMID: 30030825

[ref43] Imayoshi I. Isomura A. Harima Y. Kawaguchi K. Kori H. Miyachi H.. (2013). Oscillatory control of factors determining multipotency and fate in mouse neural progenitors. Science 342, 1203–1208. doi: 10.1126/science.1242366, PMID: 24179156

[ref44] Imayoshi I. Sakamoto M. Yamaguchi M. Mori K. Kageyama R. (2010). Essential roles of Notch signaling in maintenance of neural stem cells in developing and adult brains. J. Neurosci. 30, 3489–3498. doi: 10.1523/JNEUROSCI.4987-09.2010, PMID: 20203209 PMC6634119

[ref45] Irvin D. K. Nakano I. Paucar A. Kornblum H. I. (2004). Patterns of Jagged1, Jagged2, delta-like 1 and delta-like 3 expression during late embryonic and postnatal brain development suggest multiple functional roles in progenitors and differentiated cells. J. Neurosci. Res. 75, 330–343. doi: 10.1002/jnr.10843, PMID: 14743446

[ref46] Kakuda S. Haltiwanger R. S. (2017). Deciphering the fringe-mediated Notch code: identification of activating and inhibiting sites allowing discrimination between ligands. Dev. Cell 40, 193–201. doi: 10.1016/j.devcel.2016.12.013, PMID: 28089369 PMC5263050

[ref47] Kawaguchi D. Furutachi S. Kawai H. Hozumi K. Gotoh Y. (2013). Dll1 maintains quiescence of adult neural stem cells and segregates asymmetrically during mitosis. Nat. Commun. 4:1880. doi: 10.1038/ncomms2895, PMID: 23695674 PMC3675328

[ref48] Kawai H. Kawaguchi D. Kuebrich B. D. Kitamoto T. Yamaguchi M. Gotoh Y.. (2017). Area-specific regulation of quiescent neural stem cells by Notch3 in the adult mouse subependymal zone. J. Neurosci. 37, 11867–11880. doi: 10.1523/JNEUROSCI.0001-17.2017, PMID: 29101245 PMC6596834

[ref49] Kempermann G. Gage F. H. Aigner L. Song H. J. Curtis M. A. Thuret S.. (2018). Human adult neurogenesis: evidence and remaining questions. Cell Stem Cell 23, 25–30. doi: 10.1016/j.stem.2018.04.004, PMID: 29681514 PMC6035081

[ref50] Kjell J. Fischer-Sternjak J. Thompson A. J. Friess C. Sticco M. J. Salinas F.. (2020). Defining the adult neural stem cell niche proteome identifies key regulators of adult neurogenesis. Cell Stem Cell 26, 277–293.e8. doi: 10.1016/j.stem.2020.01.002, PMID: 32032526 PMC7005820

[ref51] Lampada A. Rolando C. Freire J. Giachino C. Parmigiani E. Engler A.. (2022). Stress and elevation of serotonin from the raphe nuclei induce Septal neuron production in adult mice. Cell Press Sneak Peek. doi: 10.2139/ssrn.4084069

[ref52] Lange C. Prenninger S. Knuckles P. Taylor V. Levin M. Calegari F. (2011). The H(+) vacuolar ATPase maintains neural stem cells in the developing mouse cortex. Stem Cells Dev. 20, 843–850. doi: 10.1089/scd.2010.0484, PMID: 21126173 PMC3128780

[ref53] Lavado A. Oliver G. (2014). Jagged1 is necessary for postnatal and adult neurogenesis in the dentate gyrus. Dev. Biol. 388, 11–21. doi: 10.1016/j.ydbio.2014.02.004, PMID: 24530424 PMC4009513

[ref54] Llorens-Bobadilla E. Zhao S. Baser A. Saiz-Castro G. Zwadlo K. Martin-Villalba A. (2015). Single-cell transcriptomics reveals a population of dormant neural stem cells that become activated upon brain injury. Cell Stem Cell 17, 329–340. doi: 10.1016/j.stem.2015.07.002, PMID: 26235341

[ref55] Lovendahl K. N. Blacklow S. C. Gordon W. R. (2018). The molecular mechanism of Notch activation. Adv. Exp. Med. Biol. 1066, 47–58. doi: 10.1007/978-3-319-89512-3_3 30030821

[ref56] Lugert S. Basak O. Knuckles P. Haussler U. Fabel K. Gotz M.. (2010). Quiescent and active hippocampal neural stem cells with distinct morphologies respond selectively to physiological and pathological stimuli and aging. Cell Stem Cell 6, 445–456. doi: 10.1016/j.stem.2010.03.017, PMID: 20452319

[ref57] Lugert E. Vogt M. Tchorz J. S. Muller M. Giachino C. Taylor V. (2012). Homeostatic neurogenesis in the adult hippocampus does not involve amplification of Ascl1(high) intermediate progenitors. Nat. Communicat. 3:670. doi: 10.1038/ncomms1670 22334073

[ref58] Matsubara S. Matsuda T. Nakashima K. (2021). Regulation of adult mammalian neural stem cells and neurogenesis by cell extrinsic and intrinsic factors. Cells 10:1145. doi: 10.3390/cells10051145, PMID: 34068607 PMC8150395

[ref59] Meloty-Kapella L. Shergill B. Kuon J. Botvinick E. Weinmaster G. (2012). Notch ligand endocytosis generates mechanical pulling force dependent on dynamin, epsins, and actin. Dev. Cell 22, 1299–1312. doi: 10.1016/j.devcel.2012.04.005, PMID: 22658936 PMC3400432

[ref60] Menn B. Garcia-Verdugo J. M. Yaschine C. Gonzalez-Perez O. Rowitch D. Alvarez-Buylla A. (2006). Origin of oligodendrocytes in the subventricular zone of the adult brain. J. Neurosci. 26, 7907–7918. doi: 10.1523/JNEUROSCI.1299-06.2006, PMID: 16870736 PMC6674207

[ref61] Merkle F. T. Fuentealba L. C. Sanders T. A. Magno L. Kessaris N. Alvarez-Buylla A. (2014). Adult neural stem cells in distinct microdomains generate previously unknown interneuron types. Nat. Neurosci. 17, 207–214. doi: 10.1038/nn.3610, PMID: 24362763 PMC4100623

[ref62] Merkle F. T. Mirzadeh Z. Alvarez-Buylla A. (2007). Mosaic organization of neural stem cells in the adult brain. Science 317, 381–384. doi: 10.1126/science.1144914, PMID: 17615304

[ref63] Merkle F. T. Tramontin A. D. Garcia-Verdugo J. M. Alvarez-Buylla A. (2004). Radial glia give rise to adult neural stem cells in the subventricular zone. Proc. Natl. Acad. Sci. U. S. A. 101, 17528–17532. doi: 10.1073/pnas.0407893101, PMID: 15574494 PMC536036

[ref64] Mirzadeh Z. Merkle F. T. Soriano-Navarro M. Garcia-Verdugo J. M. Alvarez-Buylla A. (2008). Neural stem cells confer unique pinwheel architecture to the ventricular surface in neurogenic regions of the adult brain. Cell Stem Cell 3, 265–278. doi: 10.1016/j.stem.2008.07.004, PMID: 18786414 PMC2613692

[ref65] Mizrak D. Levitin H. M. Delgado A. C. Crotet V. Yuan J. Z. Chaker Z.. (2019). Single-cell analysis of regional differences in adult V-SVZ neural stem cell lineages. Cell Rep. 26:394. doi: 10.1016/j.celrep.2018.12.044, PMID: 30625322 PMC6368857

[ref66] Musse A. A. Meloty-Kapella L. Weinmaster G. (2012). Notch ligand endocytosis: mechanistic basis of signaling activity. Semin. Cell Dev. Biol. 23, 429–436. doi: 10.1016/j.semcdb.2012.01.011, PMID: 22306180 PMC3507467

[ref67] Niola F. Zhao X. Singh D. Castano A. Sullivan R. Lauria M.. (2012). Id proteins synchronize stemness and anchorage to the niche of neural stem cells. Nat. Cell Biol. 14, 477–487. doi: 10.1038/ncb2490, PMID: 22522171 PMC3635493

[ref68] Nyfeler Y. Kirch R. D. Mantei N. Leone D. P. Radtke F. Suter U.. (2005). Jagged1 signals in the postnatal subventricular zone are required for neural stem cell self-renewal. EMBO J. 24, 3504–3515. doi: 10.1038/sj.emboj.7600816, PMID: 16163386 PMC1276174

[ref69] Obernier K. Alvarez-Buylla A. (2019). Neural stem cells: origin, heterogeneity and regulation in the adult mammalian brain. Development 146:dev156059. doi: 10.1242/dev.156059, PMID: 30777863 PMC6398449

[ref70] Obernier K. Cebrian-Silla A. Thomson M. Parraguez J. I. Anderson R. Guinto C.. (2018). Adult neurogenesis is sustained by symmetric self-renewal and differentiation. Cell Stem Cell 22, 221–234.e8. doi: 10.1016/j.stem.2018.01.003, PMID: 29395056 PMC5802882

[ref71] Oswald F. Kovall R. A. (2018). CSL-associated corepressor and coactivator complexes. Adv. Exp. Med. Biol. 1066, 279–295. doi: 10.1007/978-3-319-89512-3_14, PMID: 30030832

[ref72] Parmigiani E. Ivanek R. Rolando C. Hafen K. Turchinovich G. Lehmann F. M.. (2022). Interferon-gamma resistance and immune evasion in glioma develop via Notch-regulated co-evolution of malignant and immune cells. Dev. Cell 57, 1847–1865.e9. doi: 10.1016/j.devcel.2022.06.006, PMID: 35803280

[ref73] Parmigiani E. Taylor V. Giachino C. (2020). Oncogenic and tumor-suppressive functions of NOTCH signaling in Glioma. Cells 9:2304. doi: 10.3390/cells9102304, PMID: 33076453 PMC7602630

[ref74] Pfisterer U. Khodosevich K. (2017). Neuronal survival in the brain: neuron type-specific mechanisms. Cell Death Dis. 8:e2643. doi: 10.1038/cddis.2017.64, PMID: 28252642 PMC5386560

[ref75] Pilz G. A. Bottes S. Betizeau M. Jorg D. J. Carta S. Simons B. D.. (2018). Live imaging of neurogenesis in the adult mouse hippocampus. Science 359:658. doi: 10.1126/science.aao5056, PMID: 29439238 PMC6986926

[ref76] Rieskamp J. D. Denninger J. K. Dause T. J. (2018). Identifying the unique role of Notch3 in adult neural stem cell maintenance. J. Neurosci. 38, 3157–3159. doi: 10.1523/JNEUROSCI.3531-17.2018, PMID: 29593070 PMC5884455

[ref77] Rolando C. Erni A. Grison A. Beattie R. Engler A. Gokhale P. J.. (2016). Multipotency of adult hippocampal NSCs in vivo is restricted by Drosha/NFIB. Cell Stem Cell 19, 653–662. doi: 10.1016/j.stem.2016.07.003, PMID: 27545503

[ref78] Ryu J. R. Hong C. J. Kim J. Y. Kim E. K. Sun W. Yu S. W. (2016). Control of adult neurogenesis by programmed cell death in the mammalian brain. Mol. Brain 9:43. doi: 10.1186/s13041-016-0224-4, PMID: 27098178 PMC4839132

[ref79] Salazar J. L. Yamamoto S. (2018). Integration of Drosophila and human genetics to understand Notch signaling related diseases. Adv. Exp. Med. Biol. 1066, 141–185. doi: 10.1007/978-3-319-89512-3_8, PMID: 30030826 PMC6233323

[ref80] Sanchez-Irizarry C. Carpenter A. C. Weng A. P. Pear W. S. Aster J. C. Blacklow S. C. (2004). Notch subunit heterodimerization and prevention of ligand-independent proteolytic activation depend, respectively, on a novel domain and the LNR repeats. Mol. Cell. Biol. 24, 9265–9273. doi: 10.1128/MCB.24.21.9265-9273.2004, PMID: 15485896 PMC522238

[ref81] Schnute B. Troost T. Klein T. (2018). Endocytic trafficking of the Notch receptor. Adv. Exp. Med. Biol. 1066, 99–122. doi: 10.1007/978-3-319-89512-3_6, PMID: 30030824

[ref82] Semerci F. Choi W. T. S. Bajic A. Thakkar A. Encinas J. M. Depreux F.. (2017). Lunatic fringe-mediated Notch signaling regulates adult hippocampal neural stem cell maintenance. elife 6:e24660. doi: 10.7554/eLife.24660, PMID: 28699891 PMC5531831

[ref83] Seri B. Garcia-Verdugo J. M. Mcewen B. S. Alvarez-Buylla A. (2001). Astrocytes give rise to new neurons in the adult mammalian hippocampus. J. Neurosci. 21, 7153–7160. doi: 10.1523/JNEUROSCI.21-18-07153.2001, PMID: 11549726 PMC6762987

[ref84] Shin J. Berg D. A. Zhu Y. H. Shin J. Y. Song J. Bonaguidi M. A.. (2015). Single-cell RNA-Seq with waterfall reveals molecular cascades underlying adult neurogenesis. Cell Stem Cell 17, 360–372. doi: 10.1016/j.stem.2015.07.013, PMID: 26299571 PMC8638014

[ref85] Sierra A. Encinas J. M. Deudero J. J. Chancey J. H. Enikolopov G. Overstreet-Wadiche L. S.. (2010). Microglia shape adult hippocampal neurogenesis through apoptosis-coupled phagocytosis. Cell Stem Cell 7, 483–495. doi: 10.1016/j.stem.2010.08.014, PMID: 20887954 PMC4008496

[ref86] Sorrells S. F. Paredes M. F. Cebrian-Silla A. Sandoval K. Qi D. S. Kelley K. W.. (2018). Human hippocampal neurogenesis drops sharply in children to undetectable levels in adults. Nature 555, 377–381. doi: 10.1038/nature25975, PMID: 29513649 PMC6179355

[ref87] Sprinzak D. Blacklow S. C. (2021). Biophysics of Notch signaling. Annu. Rev. Biophys. 50, 157–189. doi: 10.1146/annurev-biophys-101920-082204, PMID: 33534608 PMC8105286

[ref88] Sprinzak D. Lakhanpal A. Lebon L. Santat L. A. Fontes M. E. Anderson G. A.. (2010). Cis-interactions between Notch and Delta generate mutually exclusive signalling states. Nature 465, 86–90. doi: 10.1038/nature08959, PMID: 20418862 PMC2886601

[ref89] Steinbuck M. P. Winandy S. (2018). A review of Notch processing with new insights into ligand-independent Notch signaling in T-cells. Front. Immunol. 9:1230. doi: 10.3389/fimmu.2018.01230, PMID: 29910816 PMC5992298

[ref90] Stump G. Durrer A. Klein A. L. Lutolf S. Suter U. Taylor V. (2002). Notch1 and its ligands Delta-like and jagged are expressed and active in distinct cell populations in the postnatal mouse brain. Mech. Dev. 114, 153–159. doi: 10.1016/S0925-4773(02)00043-6, PMID: 12175503

[ref91] Sueda R. Imayoshi I. Harima Y. Kageyama R. (2019). High Hes1 expression and resultant Ascl1 suppression regulate quiescent vs. active neural stem cells in the adult mouse brain. Genes Dev. 33, 511–523. doi: 10.1101/gad.323196.118, PMID: 30862661 PMC6499325

[ref92] Sueda R. Kageyama R. (2020). Regulation of active and quiescent somatic stem cells by Notch signaling. Develop. Growth Differ. 62, 59–66. doi: 10.1111/dgd.12626, PMID: 31489617 PMC7027910

[ref93] Tong C. K. Chen J. D. Cebrian-Silla A. Mirzadeh Z. Obernier K. Guinto C. D.. (2014). Axonal control of the adult neural stem cell niche. Cell Stem Cell 14, 500–511. doi: 10.1016/j.stem.2014.01.014, PMID: 24561083 PMC4080817

[ref94] Urban N. Blomfield I. M. Guillemot F. (2019). Quiescence of adult mammalian neural stem cells: a highly regulated rest. Neuron 104, 834–848. doi: 10.1016/j.neuron.2019.09.026, PMID: 31805262

[ref95] Urban N. Van Den Berg D. L. Forget A. Andersen J. Demmers J. A. Hunt C.. (2016). Return to quiescence of mouse neural stem cells by degradation of a proactivation protein. Science 353, 292–295. doi: 10.1126/science.aaf4802, PMID: 27418510 PMC5321528

[ref96] Van Praag H. Schinder A. F. Christie B. R. Toni N. Palmer T. D. Gage F. H. (2002). Functional neurogenesis in the adult hippocampus. Nature 415, 1030–1034. doi: 10.1038/4151030a, PMID: 11875571 PMC9284568

[ref97] Weinmaster G. Fischer J. A. (2011). Notch ligand ubiquitylation: what is it good for? Dev. Cell 21, 134–144. doi: 10.1016/j.devcel.2011.06.006, PMID: 21763614 PMC3156059

[ref98] Zhang R. R. Boareto M. Engler A. Louvi A. Giachino C. Iber D.. (2019). Id4 downstream of Notch2 maintains neural stem cell quiescence in the adult hippocampus. Cell Rep. 28:1485. doi: 10.1016/j.celrep.2019.07.014, PMID: 31390563

[ref99] Zhang R. R. Engler A. Taylor V. (2018). Notch: an interactive player in neurogenesis and disease. Cell Tissue Res. 371, 73–89. doi: 10.1007/s00441-017-2641-9, PMID: 28620760

